# Diethyl 4-(2,4-dichloro­phen­yl)-2,6-dimethyl-1,4-dihydro­pyridine-3,5-dicarboxyl­ate

**DOI:** 10.1107/S1600536810001066

**Published:** 2010-01-16

**Authors:** P. Palakshi Reddy, V. Vijayakumar, J. Suresh, T. Narasimhamurthy, P. L. Nilantha Lakshman

**Affiliations:** a Organic Chemistry Division, School of Advanced Sciences, VIT University, Vellore 632 014, India; b Department of Physics, The Madura College, Madurai 625 011, India; c Materials Research Centre, Indian Institute of Science, Bangalore 560 012, India; d Department of Food Science and Technology, University of Ruhuna, Mapalana, Kamburupitiya 81100, Sri Lanka

## Abstract

In the title compound, C_19_H_21_Cl_2_NO_4_, the dihydro­pyridine ring adopts a flattened boat conformation. The dichloro­phenyl ring is oriented almost perpendicular to the planar part of the dihydro­pyridine ring [dihedral angle = 89.1 (1)°]. An intra­molecular C—H⋯O hydrogen bond is observed. In the crystal structure, mol­ecules are linked into chains along the *b* axis by N—H⋯O hydrogen bonds

## Related literature

The dihydro­pyridine hetrocyclic ring is a common feature of various bioactive compounds such as vasodilator, anti­atherosclerotic, anti­tumor, geroprotective, hepta­protective and anti­diabetic agents, see: Salehi & Guo (2004 ▶). For ring puckering parameters, see: Cremer & Pople (1975 ▶).
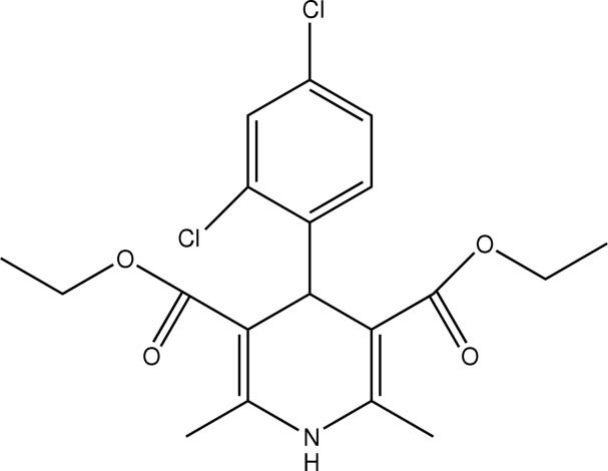


## Experimental

### 

#### Crystal data

C_19_H_21_Cl_2_NO_4_
*M* *_r_* = 398.27
Monoclinic, 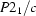

*a* = 15.928 (7) Å
*b* = 12.266 (6) Å
*c* = 10.042 (5) Å
β = 103.962 (7)°
*V* = 1903.8 (15) Å^3^
*Z* = 4
Mo *K*α radiation
μ = 0.37 mm^−1^
*T* = 293 K
0.19 × 0.16 × 0.12 mm

#### Data collection

Bruker SMART APEX CCD diffractometer
Absorption correction: multi-scan (*SADABS*; Bruker, 1998 ▶) *T*_min_ = 0.933, *T*_max_ = 0.937
20317 measured reflections
4491 independent reflections
3230 reflections with *I* > 2σ(*I*)
*R*_int_ = 0.034

#### Refinement

*R*[*F* ^2^ > 2σ(*F* ^2^)] = 0.061
*wR*(*F* ^2^) = 0.168
*S* = 1.04
4491 reflections
243 parameters
H atoms treated by a mixture of independent and constrained refinement
Δρ_max_ = 0.55 e Å^−3^
Δρ_min_ = −0.56 e Å^−3^

### 

Data collection: *SMART* (Bruker, 2001 ▶); cell refinement: *SAINT* (Bruker, 2001 ▶); data reduction: *SAINT*; program(s) used to solve structure: *SHELXS97* (Sheldrick, 2008 ▶); program(s) used to refine structure: *SHELXL97* (Sheldrick, 2008 ▶); molecular graphics: *PLATON* (Spek, 2009 ▶); software used to prepare material for publication: *SHELXL97*.

## Supplementary Material

Crystal structure: contains datablocks global, I. DOI: 10.1107/S1600536810001066/ci5001sup1.cif

Structure factors: contains datablocks I. DOI: 10.1107/S1600536810001066/ci5001Isup2.hkl

Additional supplementary materials:  crystallographic information; 3D view; checkCIF report

## Figures and Tables

**Table 1:** Hydrogen-bond geometry (Å, °)

*D*—H⋯*A*	*D*—H	H⋯*A*	*D*⋯*A*	*D*—H⋯*A*
N1—H1⋯O1^i^	0.85 (4)	2.46 (4)	3.298 (4)	169 (3)
C7—H7*C*⋯O2	0.96	2.14	2.764 (5)	122

Symmetry code: (i) 
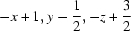
.

**Fig. 1.**
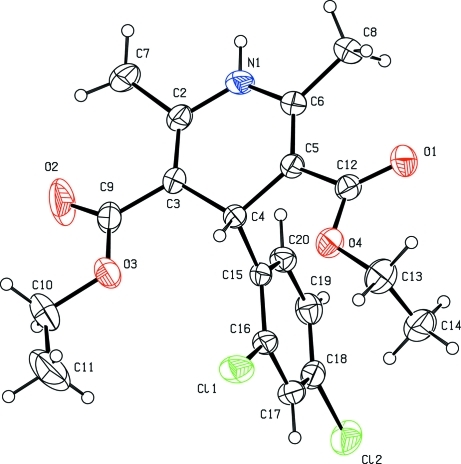
The molecular structure of the title compound, showing 30% probability displacement ellipsoids and the atom-numbering scheme.

## supplementary crystallographic information

### Comment

1,4-Dihydropyridines are identified as an important class of drugs for a longwhile. The dihydropyridine hetrocyclic ring is a common feature of various bioactive compounds such as vasodilator, antiatherosclerotic, antitumor, geroprotective, heptaprotective and antidiabetic agents (Salehi & Guo, 2004).

The molecular structure of the title compound, with the adopted atomic numbering scheme is shown in Fig. 1. The dihydropyridine ring adopts a flattened boat conformation, with atoms N1 and C4 slightly displaced out of the C2/C3/C5/C6 plane by 0.088 (4) and 0.188 (4) Å, respectively. The puckering parameters (Cremer & Pople, 1975) are: q_2_ = 0.158 (3) Å, q_3_ = -0.039 (3) Å and φ_2_ = 3(1)°. The C—C and C—N bond distances of the pyridine ring agree well with expected values. The 2,4-dichlorophenyl ring at C4 is oriented at an angle of 89.1 (1)° with respect to the C2/C3/C5/C6 plane. This near perpendicular orientation of the chlorophenyl ring to the dihydropyridine ring can be ascribed to the greater steric hinderance with the two ethylcarboxylate groups at C3 and C5. Both ethylcarboxylate side chains adopt same orientation with respect to the dihydropyridine ring. An intramolecular C7—H7C···O2 hydrogen bond is observed.

In the crystal structure, the molecules are linked into chains along the *b* axis by N—H···O hydrogen bonds (Table 1).

### Experimental

Diethyl 2,6-dimethyl-1,4-dihydro-4-2(2,6-dichlorophenyl)-3,5- pyridinedicarboxylate is prepared according to Hantzsch pyridine synthesis. 2,6-Dichlororobenzaldehyde (10 mmol, 1.76 g), ethylacetoacetate (20 mmol, 2.6 ml) and ammonium acetate (10 mmol, 0.8 g) were taken in a 1:2:1 mole ratio along with ethanol as a solvent in a flask and refluxed in steam-bath until the colour of the solution changed to reddish-orange (approximately an hour) and kept in ice cold condition to get a solid product. The product was extracted using diethyl ether and then excess solvent was distilled off. The purity of the crude product was checked through TLC and recrystallized using a acetone-benzene (3:1) solution. Single crystals of the title compound suitable for X-ray diffraction analysis were grown using a acetone-benzene (3:1) solution over a period of 2 d (yield = 68%, m.p. 413 K).

### Refinement

The amino H atom was located in a difference map and was refined isotropically. The remaining H atoms were placed in calculated positions and allowed to ride on their carrier atoms, with C-H = 0.93–0.98 Å  and *U*_iso_(H) = 1.2*U*_eq_(C) for CH and CH_2_ groups and *U*_iso_(H) = 1.5*U*_eq_(C) for CH_3_ groups.

### Crystal data

**Table:**

C_19_H_21_Cl_2_NO_4_	*F*(000) = 832
*M**_r_* = 398.27	*D*_x_ = 1.389 Mg m^−^^3^
Monoclinic, *P*2_1_/*c*	Mo *K*α radiation, λ = 0.71073 Å
Hall symbol: -P 2ybc	Cell parameters from 25 reflections
*a* = 15.928 (7) Å	θ = 2–28°
*b* = 12.266 (6) Å	µ = 0.37 mm^−^^1^
*c* = 10.042 (5) Å	*T* = 293 K
β = 103.962 (7)°	Block, colourless
*V* = 1903.8 (15) Å^3^	0.19 × 0.16 × 0.12 mm
*Z* = 4	

### Data collection

**Table:**

Bruker SMART APEX CCD diffractometer	4491 independent reflections
Radiation source: fine-focus sealed tube	3230 reflections with *I* > 2σ(*I*)
graphite	*R*_int_ = 0.034
ω scans	θ_max_ = 28.0°, θ_min_ = 2.1°
Absorption correction: multi-scan (*SADABS*; Bruker, 1998)	*h* = −20→20
*T*_min_ = 0.933, *T*_max_ = 0.937	*k* = −16→15
20317 measured reflections	*l* = −13→13

### Refinement

**Table:**

Refinement on *F*^2^	Primary atom site location: structure-invariant direct methods
Least-squares matrix: full	Secondary atom site location: difference Fourier map
*R*[*F*^2^ > 2σ(*F*^2^)] = 0.061	Hydrogen site location: inferred from neighbouring sites
*wR*(*F*^2^) = 0.168	H atoms treated by a mixture of independent and constrained refinement
*S* = 1.03	*w* = 1/[σ^2^(*F*_o_^2^) + (0.0754*P*)^2^ + 1.087*P*] where *P* = (*F*_o_^2^ + 2*F*_c_^2^)/3
4491 reflections	(Δ/σ)_max_ = 0.001
243 parameters	Δρ_max_ = 0.55 e Å^−^^3^
0 restraints	Δρ_min_ = −0.56 e Å^−^^3^

### Special details

**Table:**

Geometry. All e.s.d.'s (except the e.s.d. in the dihedral angle between two l.s. planes) are estimated using the full covariance matrix. The cell e.s.d.'s are taken into account individually in the estimation of e.s.d.'s in distances, angles and torsion angles; correlations between e.s.d.'s in cell parameters are only used when they are defined by crystal symmetry. An approximate (isotropic) treatment of cell e.s.d.'s is used for estimating e.s.d.'s involving l.s. planes.
Refinement. Refinement of *F*^2^ against ALL reflections. The weighted *R*-factor *wR* and goodness of fit *S* are based on *F*^2^, conventional *R*-factors *R* are based on *F*, with *F* set to zero for negative *F*^2^. The threshold expression of *F*^2^ > σ(*F*^2^) is used only for calculating *R*-factors(gt) *etc*. and is not relevant to the choice of reflections for refinement. *R*-factors based on *F*^2^ are statistically about twice as large as those based on *F*, and *R*- factors based on ALL data will be even larger.

### Fractional atomic coordinates and isotropic or equivalent isotropic displacement parameters (Å^2^)

**Table:**

	*x*	*y*	*z*	*U*_iso_*/*U*_eq_	
H1	0.457 (2)	−0.188 (3)	0.674 (4)	0.077 (10)*	
C2	0.36076 (17)	−0.1934 (2)	0.5152 (3)	0.0498 (6)	
C3	0.30628 (16)	−0.13017 (19)	0.4249 (3)	0.0443 (5)	
C4	0.30510 (14)	−0.00633 (18)	0.4398 (2)	0.0385 (5)	
H4	0.3047	0.0264	0.3506	0.046*	
C5	0.38599 (15)	0.03294 (19)	0.5438 (2)	0.0414 (5)	
C6	0.43734 (15)	−0.0368 (2)	0.6316 (3)	0.0458 (6)	
C7	0.3671 (2)	−0.3158 (2)	0.5144 (4)	0.0724 (9)	
H7A	0.3368	−0.3457	0.5778	0.109*	
H7B	0.4268	−0.3370	0.5411	0.109*	
H7C	0.3418	−0.3426	0.4238	0.109*	
C8	0.51522 (18)	−0.0086 (3)	0.7436 (3)	0.0615 (7)	
H8A	0.5467	0.0493	0.7133	0.092*	
H8B	0.5518	−0.0715	0.7656	0.092*	
H8C	0.4970	0.0145	0.8236	0.092*	
C9	0.2429 (2)	−0.1811 (2)	0.3105 (3)	0.0576 (7)	
C10	0.1394 (2)	−0.1567 (3)	0.1037 (3)	0.0834 (11)	
H10A	0.1663	−0.2102	0.0562	0.100*	
H10B	0.0947	−0.1929	0.1378	0.100*	
C11	0.1024 (4)	−0.0724 (5)	0.0120 (5)	0.146 (2)	
H11A	0.0756	−0.0199	0.0593	0.219*	
H11B	0.0598	−0.1027	−0.0631	0.219*	
H11C	0.1468	−0.0375	−0.0224	0.219*	
C12	0.40564 (15)	0.1502 (2)	0.5500 (3)	0.0465 (6)	
C13	0.3635 (2)	0.3178 (2)	0.4302 (4)	0.0708 (9)	
H13A	0.3527	0.3407	0.3351	0.085*	
H13B	0.4207	0.3428	0.4773	0.085*	
C14	0.2986 (2)	0.3668 (3)	0.4941 (4)	0.0847 (11)	
H14A	0.2421	0.3411	0.4482	0.127*	
H14B	0.3006	0.4447	0.4866	0.127*	
H14C	0.3108	0.3465	0.5892	0.127*	
C15	0.22400 (14)	0.03096 (17)	0.4827 (2)	0.0382 (5)	
C16	0.16297 (16)	0.10357 (19)	0.4097 (3)	0.0442 (5)	
C17	0.09232 (16)	0.1380 (2)	0.4559 (3)	0.0529 (7)	
H17	0.0531	0.1877	0.4057	0.064*	
C18	0.08165 (16)	0.0971 (2)	0.5769 (3)	0.0540 (7)	
C19	0.13873 (18)	0.0238 (2)	0.6521 (3)	0.0554 (7)	
H19	0.1301	−0.0040	0.7339	0.066*	
C20	0.20941 (16)	−0.0081 (2)	0.6045 (3)	0.0456 (6)	
H20	0.2485	−0.0574	0.6559	0.055*	
N1	0.42086 (15)	−0.14648 (18)	0.6216 (3)	0.0549 (6)	
O1	0.45504 (13)	0.19882 (16)	0.6420 (2)	0.0648 (5)	
O2	0.2248 (3)	−0.2760 (2)	0.3005 (3)	0.1241 (13)	
O3	0.20393 (13)	−0.11059 (17)	0.2178 (2)	0.0639 (5)	
O4	0.36015 (13)	0.19982 (14)	0.4368 (2)	0.0577 (5)	
Cl1	0.17106 (5)	0.15619 (6)	0.25211 (7)	0.0651 (2)	
Cl2	−0.00735 (5)	0.13933 (8)	0.63631 (10)	0.0800 (3)	

### Atomic displacement parameters (Å^2^)

**Table:**

	*U*^11^	*U*^22^	*U*^33^	*U*^12^	*U*^13^	*U*^23^
C2	0.0523 (14)	0.0362 (12)	0.0598 (16)	0.0037 (11)	0.0118 (12)	−0.0033 (11)
C3	0.0485 (13)	0.0359 (12)	0.0476 (13)	0.0012 (10)	0.0099 (11)	−0.0058 (10)
C4	0.0407 (12)	0.0335 (11)	0.0393 (12)	0.0007 (9)	0.0056 (9)	0.0004 (9)
C5	0.0400 (12)	0.0385 (12)	0.0454 (13)	0.0003 (10)	0.0098 (10)	−0.0031 (10)
C6	0.0406 (12)	0.0461 (13)	0.0486 (14)	0.0025 (10)	0.0066 (10)	−0.0023 (11)
C7	0.076 (2)	0.0376 (14)	0.095 (2)	0.0127 (14)	0.0044 (18)	0.0006 (15)
C8	0.0507 (15)	0.0642 (18)	0.0604 (17)	0.0047 (13)	−0.0048 (13)	−0.0028 (14)
C9	0.0729 (18)	0.0470 (15)	0.0500 (15)	−0.0009 (13)	0.0093 (13)	−0.0108 (12)
C10	0.078 (2)	0.104 (3)	0.0564 (19)	−0.020 (2)	−0.0069 (16)	−0.0176 (19)
C11	0.149 (5)	0.130 (4)	0.108 (4)	−0.009 (4)	−0.069 (3)	0.005 (3)
C12	0.0417 (13)	0.0409 (13)	0.0578 (15)	−0.0012 (10)	0.0135 (11)	−0.0022 (11)
C13	0.087 (2)	0.0406 (15)	0.086 (2)	−0.0071 (15)	0.0233 (18)	0.0076 (15)
C14	0.079 (2)	0.0552 (19)	0.114 (3)	0.0075 (17)	0.012 (2)	−0.0121 (19)
C15	0.0391 (11)	0.0308 (10)	0.0412 (12)	−0.0013 (9)	0.0032 (9)	−0.0027 (9)
C16	0.0448 (13)	0.0369 (12)	0.0470 (13)	0.0020 (10)	0.0034 (10)	0.0019 (10)
C17	0.0440 (13)	0.0440 (14)	0.0667 (17)	0.0065 (11)	0.0052 (12)	−0.0034 (12)
C18	0.0414 (13)	0.0506 (15)	0.0718 (18)	−0.0042 (11)	0.0168 (12)	−0.0209 (13)
C19	0.0571 (16)	0.0606 (17)	0.0506 (15)	−0.0094 (13)	0.0170 (12)	−0.0063 (12)
C20	0.0457 (13)	0.0429 (13)	0.0461 (13)	0.0005 (10)	0.0066 (10)	0.0036 (10)
N1	0.0532 (13)	0.0411 (12)	0.0615 (14)	0.0088 (10)	−0.0033 (11)	0.0063 (10)
O1	0.0614 (12)	0.0495 (11)	0.0764 (14)	−0.0127 (9)	0.0026 (10)	−0.0115 (10)
O2	0.192 (3)	0.0500 (14)	0.095 (2)	−0.0218 (17)	−0.035 (2)	−0.0179 (13)
O3	0.0652 (12)	0.0620 (12)	0.0538 (11)	−0.0057 (10)	−0.0063 (9)	−0.0078 (9)
O4	0.0673 (12)	0.0372 (9)	0.0651 (12)	−0.0024 (8)	0.0089 (9)	0.0024 (8)
Cl1	0.0712 (5)	0.0637 (4)	0.0564 (4)	0.0147 (4)	0.0075 (3)	0.0212 (3)
Cl2	0.0531 (4)	0.0868 (6)	0.1078 (7)	−0.0043 (4)	0.0343 (4)	−0.0335 (5)

### Geometric parameters (Å, °)

**Table:**

C2—C3	1.341 (4)	C11—H11A	0.96
C2—N1	1.376 (3)	C11—H11B	0.96
C2—C7	1.504 (4)	C11—H11C	0.96
C3—C9	1.473 (4)	C12—O1	1.216 (3)
C3—C4	1.527 (3)	C12—O4	1.338 (3)
C4—C15	1.527 (3)	C13—O4	1.450 (3)
C4—C5	1.528 (3)	C13—C14	1.470 (5)
C4—H4	0.98	C13—H13A	0.97
C5—C6	1.353 (3)	C13—H13B	0.97
C5—C12	1.470 (3)	C14—H14A	0.96
C6—N1	1.370 (3)	C14—H14B	0.96
C6—C8	1.500 (4)	C14—H14C	0.96
C7—H7A	0.96	C15—C20	1.384 (3)
C7—H7B	0.96	C15—C16	1.390 (3)
C7—H7C	0.96	C16—C17	1.383 (4)
C8—H8A	0.96	C16—Cl1	1.743 (3)
C8—H8B	0.96	C17—C18	1.363 (4)
C8—H8C	0.96	C17—H17	0.93
C9—O2	1.198 (4)	C18—C19	1.368 (4)
C9—O3	1.312 (3)	C18—Cl2	1.744 (3)
C10—C11	1.414 (6)	C19—C20	1.382 (4)
C10—O3	1.456 (3)	C19—H19	0.93
C10—H10A	0.97	C20—H20	0.93
C10—H10B	0.97	N1—H1	0.85 (4)
			
C3—C2—N1	119.9 (2)	C10—C11—H11C	109.5
C3—C2—C7	127.4 (3)	H11A—C11—H11C	109.5
N1—C2—C7	112.7 (2)	H11B—C11—H11C	109.5
C2—C3—C9	119.5 (2)	O1—C12—O4	122.7 (2)
C2—C3—C4	122.0 (2)	O1—C12—C5	127.2 (2)
C9—C3—C4	118.4 (2)	O4—C12—C5	110.1 (2)
C3—C4—C15	110.91 (19)	O4—C13—C14	110.5 (3)
C3—C4—C5	110.63 (19)	O4—C13—H13A	109.5
C15—C4—C5	110.11 (19)	C14—C13—H13A	109.5
C3—C4—H4	108.4	O4—C13—H13B	109.5
C15—C4—H4	108.4	C14—C13—H13B	109.5
C5—C4—H4	108.4	H13A—C13—H13B	108.1
C6—C5—C12	120.1 (2)	C13—C14—H14A	109.5
C6—C5—C4	121.6 (2)	C13—C14—H14B	109.5
C12—C5—C4	118.2 (2)	H14A—C14—H14B	109.5
C5—C6—N1	119.9 (2)	C13—C14—H14C	109.5
C5—C6—C8	127.1 (2)	H14A—C14—H14C	109.5
N1—C6—C8	113.0 (2)	H14B—C14—H14C	109.5
C2—C7—H7A	109.5	C20—C15—C16	116.2 (2)
C2—C7—H7B	109.5	C20—C15—C4	118.6 (2)
H7A—C7—H7B	109.5	C16—C15—C4	125.1 (2)
C2—C7—H7C	109.5	C17—C16—C15	122.8 (2)
H7A—C7—H7C	109.5	C17—C16—Cl1	115.87 (19)
H7B—C7—H7C	109.5	C15—C16—Cl1	121.4 (2)
C6—C8—H8A	109.5	C18—C17—C16	118.3 (2)
C6—C8—H8B	109.5	C18—C17—H17	120.9
H8A—C8—H8B	109.5	C16—C17—H17	120.9
C6—C8—H8C	109.5	C17—C18—C19	121.6 (2)
H8A—C8—H8C	109.5	C17—C18—Cl2	118.8 (2)
H8B—C8—H8C	109.5	C19—C18—Cl2	119.6 (2)
O2—C9—O3	121.2 (3)	C18—C19—C20	118.9 (3)
O2—C9—C3	125.7 (3)	C18—C19—H19	120.5
O3—C9—C3	113.1 (2)	C20—C19—H19	120.5
C11—C10—O3	109.4 (3)	C19—C20—C15	122.2 (2)
C11—C10—H10A	109.8	C19—C20—H20	118.9
O3—C10—H10A	109.8	C15—C20—H20	118.9
C11—C10—H10B	109.8	C6—N1—C2	123.6 (2)
O3—C10—H10B	109.8	C6—N1—H1	117 (2)
H10A—C10—H10B	108.2	C2—N1—H1	118 (2)
C10—C11—H11A	109.5	C9—O3—C10	115.2 (3)
C10—C11—H11B	109.5	C12—O4—C13	118.3 (2)
H11A—C11—H11B	109.5		

### Hydrogen-bond geometry (Å, °)

**Table:**

*D*—H···*A*	*D*—H	H···*A*	*D*···*A*	*D*—H···*A*
N1—H1···O1^i^	0.85 (4)	2.46 (4)	3.298 (4)	169 (3)
C7—H7C···O2	0.96	2.14	2.764 (5)	122

Symmetry codes: (i) −*x*+1, *y*−1/2, −*z*+3/2.
